# Intraductal Papillary Neoplasm of the Bile Duct Mimicking a Cholangiocarcinoma

**DOI:** 10.7759/cureus.73226

**Published:** 2024-11-07

**Authors:** José Couto, Aires Martins, João Mendes, Ana Cristina Rodrigues

**Affiliations:** 1 General Surgery, Local Health Unit of Alto Minho, Viana do Castelo, PRT

**Keywords:** benign lesion, dilated common bile duct, extrahepatic cholangiocarcinoma, extrahepatic cholestasis, hepato-pancreato-biliary surgery

## Abstract

The intraductal papillary neoplasm of the bile duct is a rare tumor considered one of the precursor lesions of cholangiocarcinoma. It is characterized by papillary growth within the bile duct lumen, occurring anywhere in the biliary tree. We report a case of a 70-year-old man who presented with a six-month history of weight loss and occasional choluria. An abdominal ultrasound showed a marked dilatation of the biliary tree with a polypoid intraluminal lesion. Computed tomography and magnetic resonance imaging confirmed the presence of these findings and did not reveal any secondary lesions. The patient underwent a cephalic duodenopancreatectomy yielding a diagnosis of intraductal papillary neoplasm with high-grade dysplasia.

## Introduction

There are two main types of pre-invasive neoplasms of bile ducts preceding cholangiocarcinoma: biliary intraepithelial neoplasm (BilIN) and intraductal papillary neoplasm of the bile duct (IPNB) [1-4]. IPNB is a rare disease entity with a prevalence of 4% to 15% among bile duct tumors, and it is more common in eastern countries [5]. Although the specific etiology is uncertain, IPNB is known to have two major risk factors: hepatolithiasis and clonorchiasis [6].

Clinically, IPNB could be divided into type 1 and type 2. The former typically evolves at the level of intrahepatic bile ducts and type 2 with a more complex histologic papillary architecture develops inside the extrahepatic bile duct [5].

It typically affects middle-aged and elderly adults [7,8]. It is characterized by an intraductal polypoid growing tumor of the intrahepatic or extrahepatic bile ducts [1,4].

The most common clinical features are intermittent right-upper-quadrant abdominal pain and jaundice or recurrent acute cholangitis [5]. IPNBs may undergo sequential progression from low-grade to high-grade and invasive adenocarcinoma [9]. All patients with this pre-malignant lesion should be considered for surgical resection due to its recurrent symptoms and potential for malignancy [7,10].

This article was previously presented as a poster at the 42nd Congress of the European Society of Surgical Oncology from 25 to 27 October 2023 in Florence, Italy.

## Case presentation

**Figure 1:**
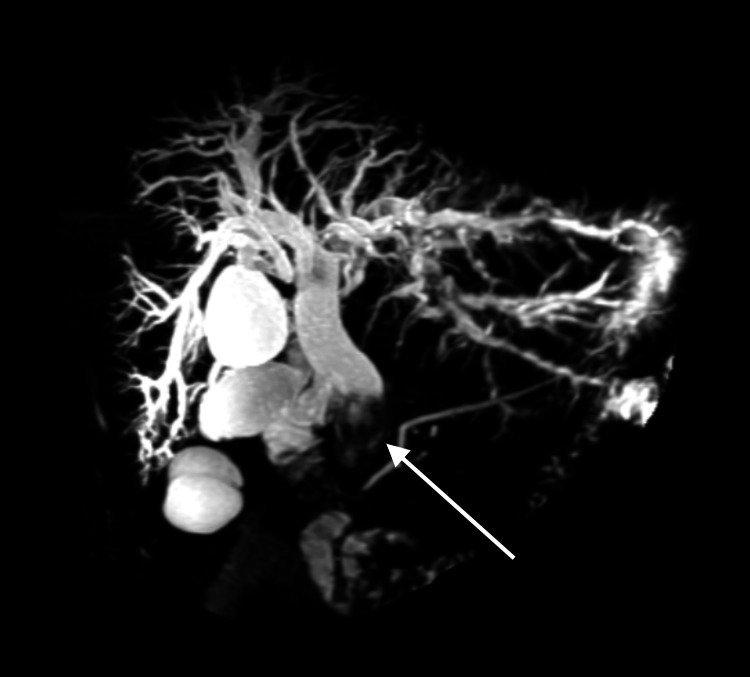
Coronal MRCP maximum intensity projection (MIP) reformation. Coronal magnetic resonance cholangiography (MRCP) maximum intensity projection (MIP) reformation demonstrates dilated intra- and extrahepatic bile ducts, along with slight prominence of the main pancreatic duct. An endoluminal lesion is identified in the distal common bile duct (arrow).

**Figure 2:**
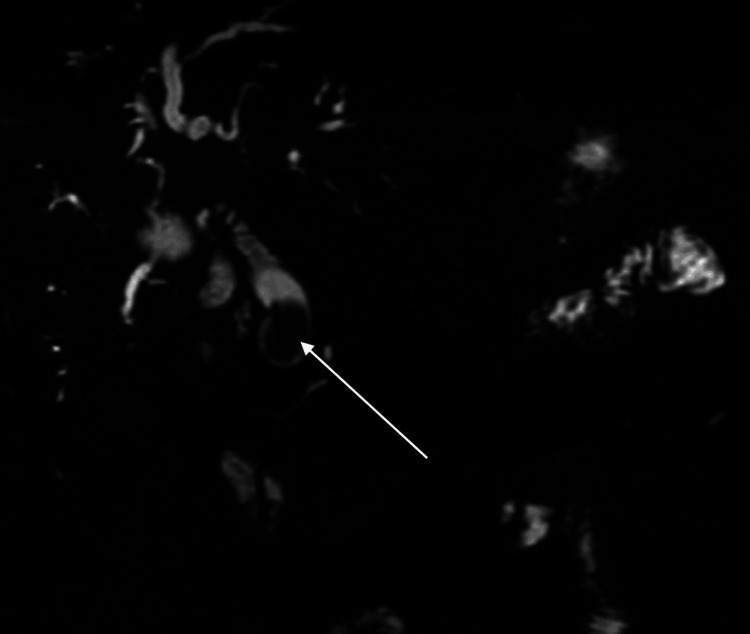
Coronal MRCP thin slice oriented to the common bile duct. Coronal magnetic resonance cholangiography (MRCP) image reveals a dilated common bile duct with an endoluminal lesion located in the distal portion of the duct (arrow).

**Figure 3:**
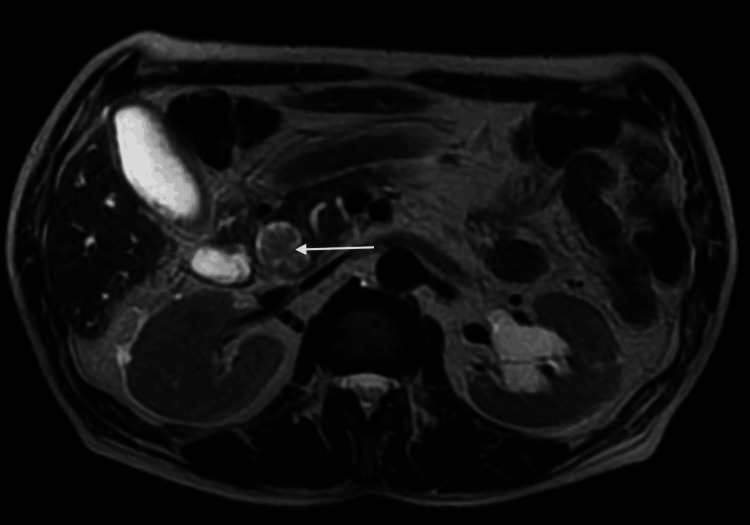
Axial T2-weighted image. Axial T2-weighted image depicts an intermediate signal lesion within the distal common bile duct (arrow). The tumor is confined to the common bile duct with an intraluminal growth. There is no sign of invasion of adjacent structures.

**Figure 4:**
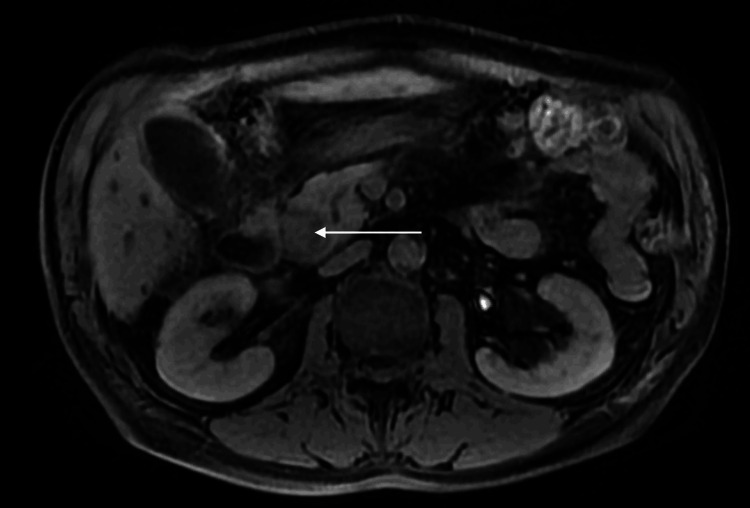
Axial T1-weighted fat-saturated image. Axial T1-weighted fat-saturated image acquired after intravenous gadolinium administration during the late venous phase displays a hyperintense lesion in the distal common bile duct (arrow).

**Figure 5:**
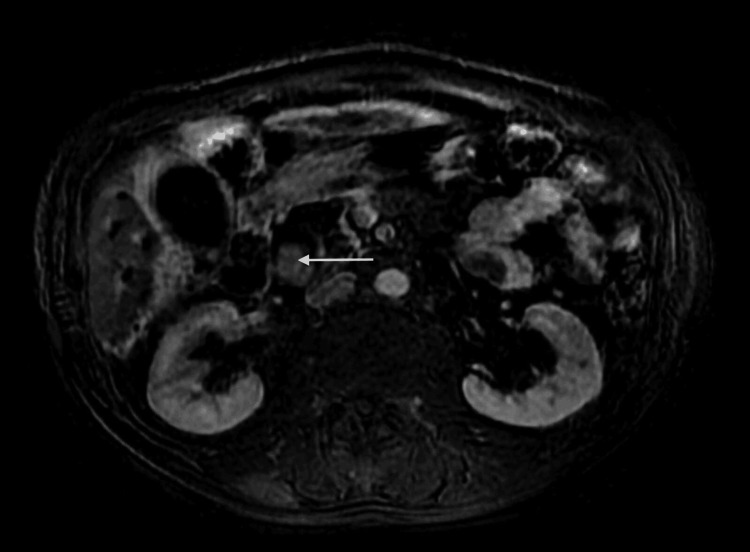
Post-contrast T1-weighted fat-saturated subtraction imaging. Post-contrast T1-weighted fat-saturated subtraction imaging confirms the enhancement of the lesion in the distal common bile duct (arrow).

We report a case of a 70-year-old man with a six-month history of significant weight loss (15%) associated with episodic choluria. Abdominal ultrasound (US) was obtained, exhibiting marked dilation of intrahepatic bile ducts and the common bile duct (CBD) reaching a caliber of 22 mm. Several confluent solid-type hyperechogenic images were observed, the largest of which had a size of 21 mm. A further computed tomography (CT) scan of the abdomen confirmed a marked dilatation of the CBD (maximum diameter of 25 mm) upstream from an endoluminal lesion with an irregular shape. No ascites or enlarged lymph nodes were observed in the abdominal cavity or retroperitoneum. The study was then extended with magnetic resonance cholangiography (MRCP). It showed a polypoid lesion in the lumen of the distal third of the CBD with 29 mm in diameter. Some hepatobiliary cysts were observed, the largest of which was 35 mm in diameter (Figures 1-5).

The liver biochemistry at presentation was as follows: total bilirubin = 1.63 mg/dL; direct bilirubin = 0.95 mg/dL; alkaline phosphatase = 1408 mg/dL; alanine aminotransferase = 74 U/L; aspartate aminotransferase = 49 U/L; gamma-glutamyl transpeptidase = 1621 U/L. Tumor markers, cancer antigen 19-9 (CA 19-9) and carcinoembryonic antigen (CEA), were within normal range. Based on these findings, our patient was discussed in the hepatobiliary multidisciplinary group and surgical resection was decided. The patient underwent an open cephalic duodenopancreatectomy (CDP).

**Figure 6:**
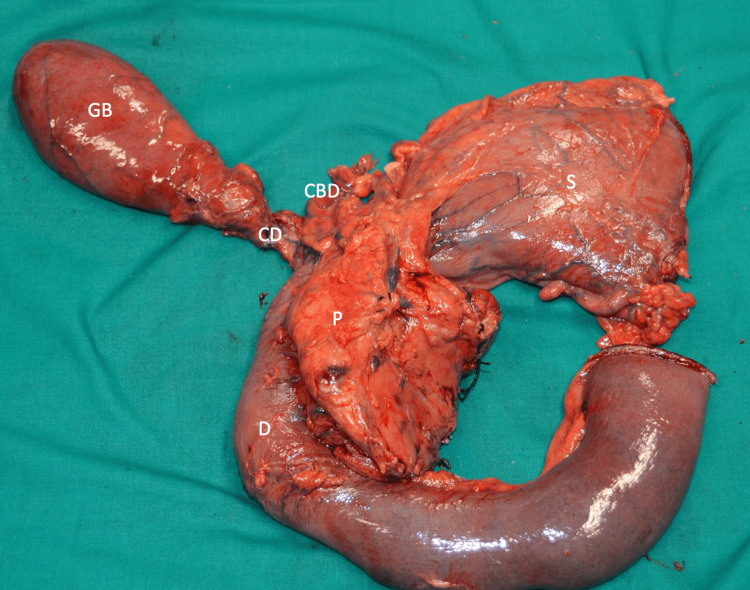
Cephalic duodenopancreatectomy. Gross examination of the surgically resected specimen. CBD, common bile duct; CD: cystic duct; D: duodenum; GB, gallbladder; P: pancreas.

**Figure 7:**
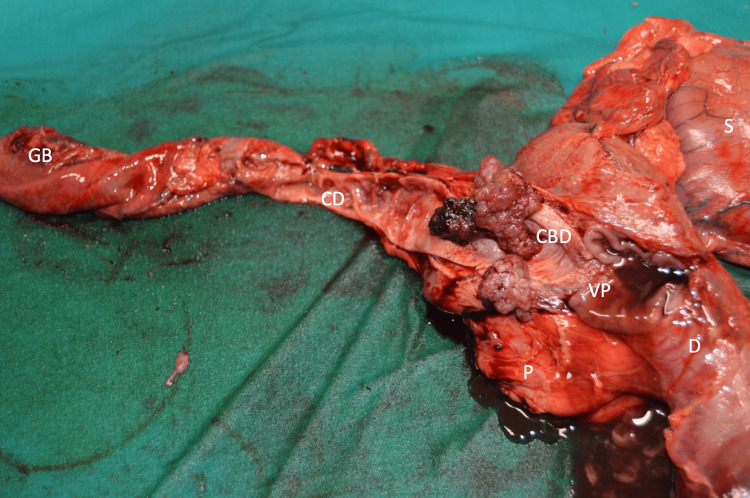
Gross features: polypoid lesion in the distal common bile duct, 2.8 cm, proximal from the Vater papilla, with a brownish surface, measuring 2.5 x 2 cm. CBD, common bile duct; CD: cystic duct; D: duodenum; GB, gallbladder; P: pancreas; S: stomach; VP: Vater papilla.

**Figure 8:**
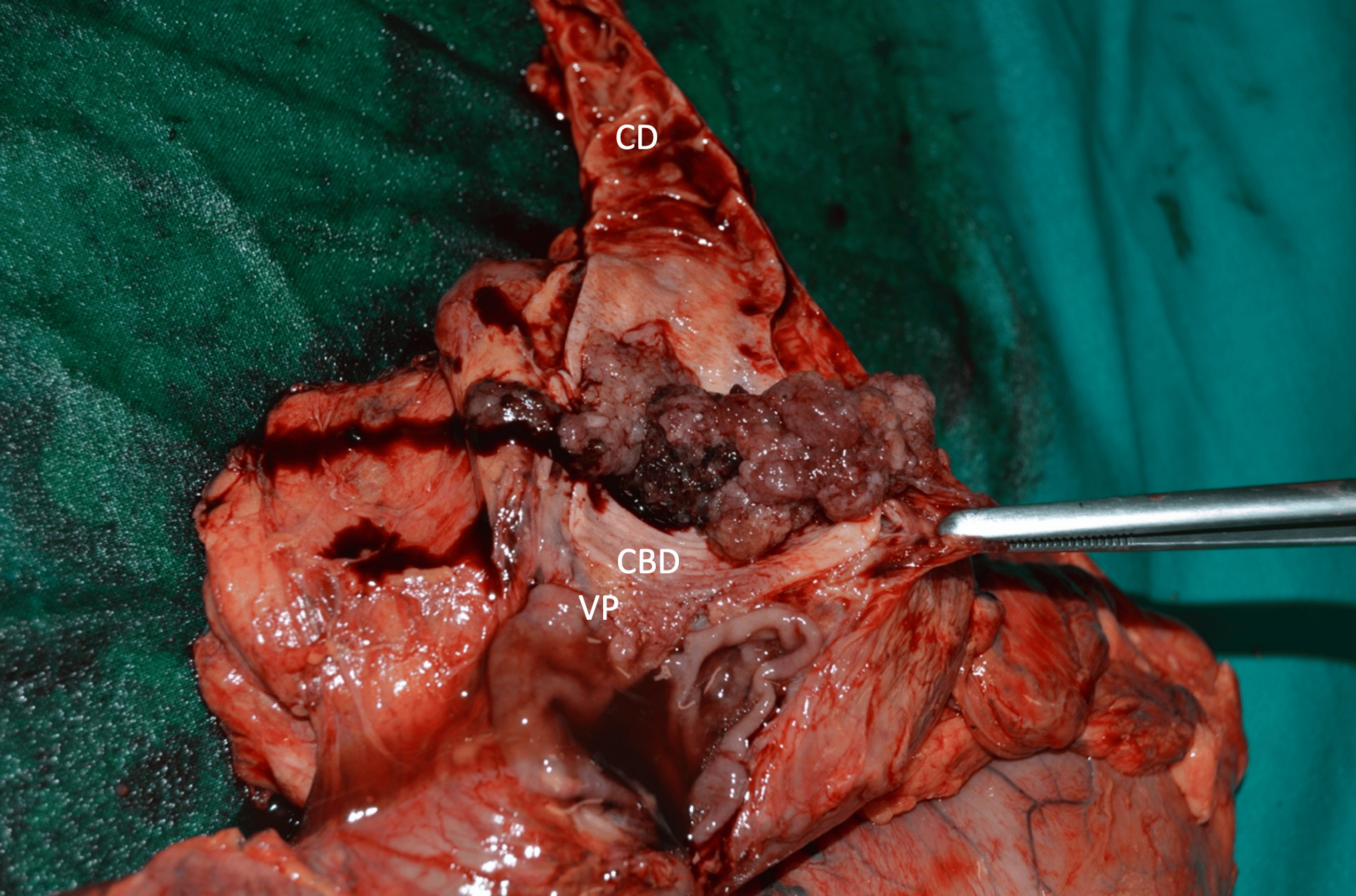
Gross features: polypoid lesion in the distal common bile duct, completely occluding it, not seen from this perspective. CBD, common bile duct; CD: cystic duct; VP: Vater papilla.

**Figure 9:**
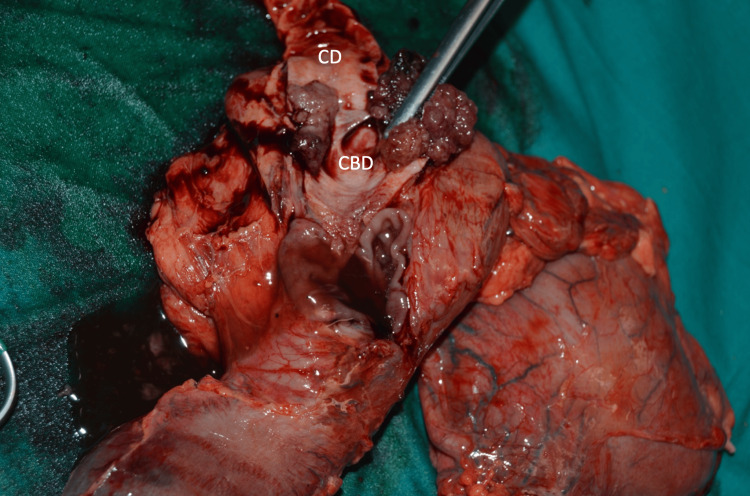
Observation of the main bile duct ostium after dislodging the lesion. CBD: common bile duct; CD: cystic duct.

Surgery was uneventful with no blood transfusion required. The operative time was 660 minutes, and the total blood loss was 300 mL. Intraoperatively, a frozen section of the bile duct stump was performed to confirm a negative margin, given the tendency of these tumors to longitudinal spread along the bile duct and their multifocal nature [11]. The postoperative period was marked by a pancreatic fistula grade B [12] (surgical drain left in place for three weeks). On gross examination, in distal CBD, a protruding lesion was identified, with a polypoid/arboriform configuration measuring 25 x 20 mm, causing dilation of the upstream CBD (Figures 6-9).

**Figure 10:**
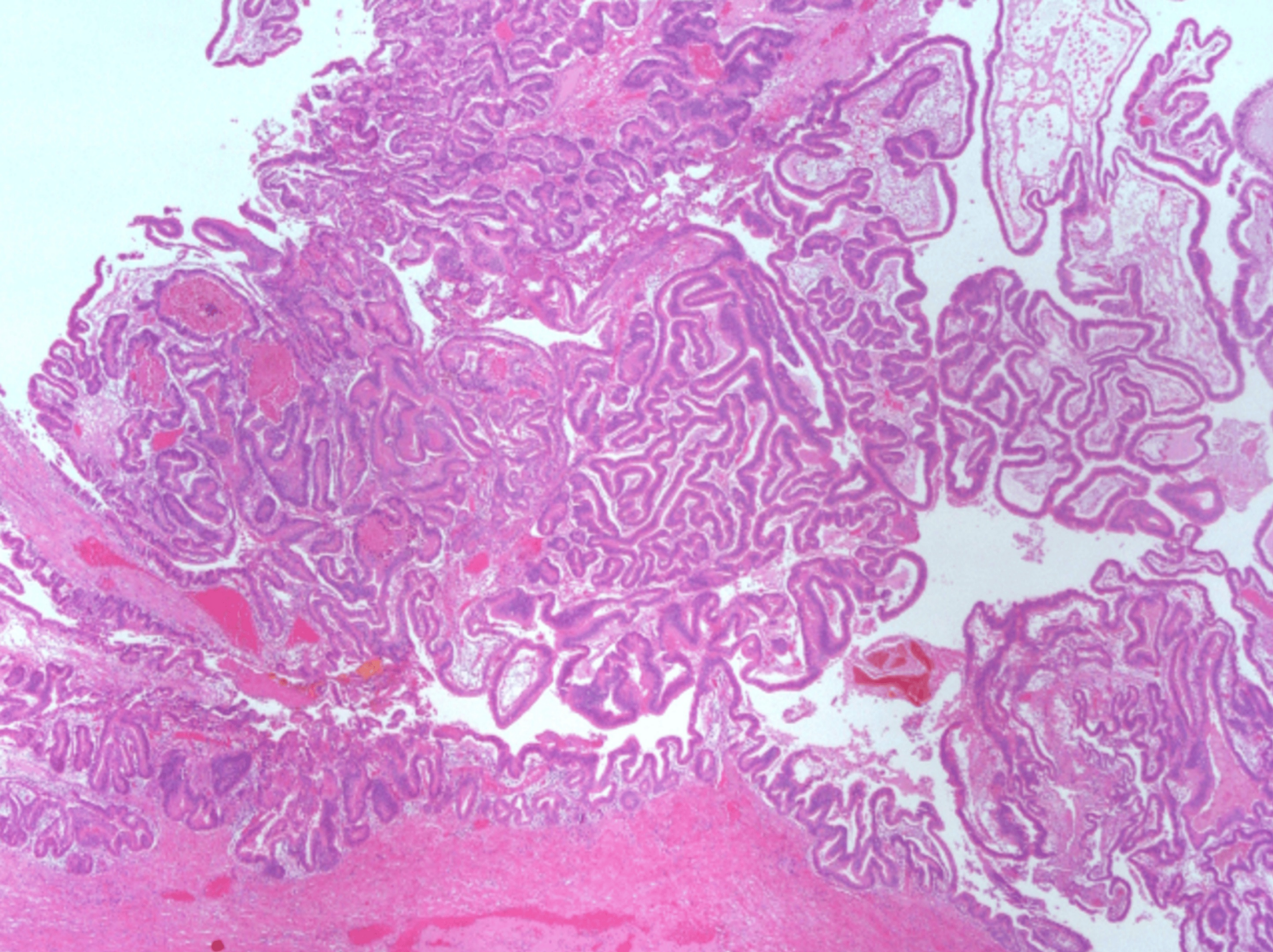
Histological features of intraductal papillary neoplasm of the bile duct (IPNB).

Microscopic examination showed a lesion composed of fibrovascular cores covered by cuboidal to columnar atypical epithelial, lacking ovarian-type stroma and invasion (Figure 10).

Immunohistochemical staining showed positivity for cytokeratin 7 (CK7), cytokeratin 20 (CK20), and caudal homeobox 2 (CDX2). All these findings were consistent with an IPNB with high-grade dysplasia, intestinal subtype. BilIN was identified in the surrounding bile duct mucosa. The resection margins were free of dysplasia (R0 resection). A total of 22 lymph nodes were harvested, and all were negative for metastasis.

## Discussion

IPNB was adopted in the 2010 World Health Organization classification as a precursor of cholangiocarcinoma. This lesion is characterized by the exophytic proliferation of biliary epithelium on fibrovascular stalks within the bile duct, which gradually transforms from adenoma to adenocarcinoma [13,14].

IPNB resembles the intraductal papillary mucinous neoplasm (IPMN) of the pancreas, with both showing comparable pathogenetic processes, the same histological subtypes, and the association with mucin hypersecretion [15].

IPMNs of the pancreas are also pre-invasive intraductal papillary neoplasms and are characterized by the intramucosal spread of neoplastic epithelia followed by invasive carcinoma [15].

There are two types of IPNB, type 1 IPNB is histologically similar to IPMN, and typically develops in the intrahepatic bile ducts, while type 2 IPNB has a more complex histological architecture with irregular papillary branching or with foci of solid-tubular components and commonly involves the extrahepatic bile ducts [16]. Therefore, this IPNB is considered a type 2.

There are four histological subtypes based on their epithelial cell lineages: intestinal, gastric, pancreatobiliary, and oncocytic [2,7,9,17]. The pancreatobiliary subtype has the highest potential for invasion [10].

IPNB is often asymptomatic and detected incidentally. When symptoms do occur, they are typically due to intrinsic biliary obstruction, which can lead to obstructive jaundice or cholangitis.

In our case, the patient presented with intermittent choluria and weight loss. The liver enzymes were slightly elevated. These findings taken together prompted his primary care physician to request an abdominal US that unveiled the neoformative lesion in the CBD. The treatment of choice for patients without distant metastasis is surgical resection to alleviate the biliary obstruction symptoms and treat or prevent malignancy. For our patient, the most suitable surgery was CDP. Histological examination of the tumor revealed no invasive component. These polypoid tumors grow in the lumen of the duct and lead to biliary obstruction early in the course of the disease. Consequently, these tumors have the highest resectability rates with a better prognosis than cholangiocarcinoma.

Notwithstanding, IPNB has a risk of recurrence that cannot be ignored. A recent review article reported that the cumulative one-, three-, and five-year overall survival (OS) rates for IPNB were 97.2%, 89.6%, and 80.9%, respectively [18]. For this reason, these patients need a follow-up, which is not yet fully established.

Our patient was followed up every six months for the first year after surgery. Blood tests, CT, and MRI were used for examinations.

## Conclusions

IPNB is an uncommon tumor of the biliary epithelium that needs to be distinguished from cholangiopathies and cholangiocarcinoma to facilitate appropriate surgical interventions at an early stage. This is a potentially curable disease, requiring a R0 resection and a close follow-up. Due to the increasing access to radiological imaging methods, the incidence of this pre-invasive neoplasm of the bile duct will tend to increase.
